# Material Fracturing and Failure Simulation Datasets

**DOI:** 10.1038/s41597-025-06412-8

**Published:** 2025-12-13

**Authors:** Ryley G. Hill, Kai Gao, Aleksandra Pachalieva, Agnese Marcato, Xiaoyu Wang, Pascal Grosset, Esteban Rougier, Zhou Lei, Javier E. Santos, Vinamra Agrawal, Qinjun Kang, Jeffrey D. Hyman, Abigail Hunter, Christine M. Sweeney, Nathan DeBardeleben, Earl Lawrence, Hari Viswanathan, Daniel O’Malley

**Affiliations:** 1 National Security Earth Science Group, Earth and Environmental Sciences Division, Los Alamos National Laboratory, Los Alamos, NM 87545 USA; 2 Energy and Natural Resources Security Group, Earth and Environmental Sciences Division, Los Alamos National Laboratory, Los Alamos, NM 87545 USA; 3 Statistical Sciences Group, Computer, Computational and Statistical Sciences Division, Los Alamos National Laboratory, Los Alamos, NM 87545 USA; 4 Materials and Physical Data Group, X Computational Physics Division, Los Alamos National Laboratory, Los Alamos, NM 87545 USA; 5 Applied Computer Science Group, Computing and Artificial Intelligence Division, Los Alamos National Laboratory, Los Alamos, NM 87545 USA; 6 High Performance Computing Design Group, High Performance Computing Division, Los Alamos National Laboratory, Los Alamos, NM 87545 USA

**Keywords:** Materials science, Databases

## Abstract

Fracturing is a fundamental physics phenomena with broad relevance across multiple domains, ranging from infrastructure integrity, aerospace durability, reservoir production, and seismic events. We present a diverse dataset of simulated fracture evolution and material failure generated from two numerical solvers: the phase-field method and the combined finite-discrete element method (FDEM). These solvers differ in formulation, physical fidelity, and computational efficiency. The dataset includes five materials: PBX, anisotropic shale, tungsten, aluminum, and steel. For each, phase-field simulations span 400,000 cases: 200,000 under uniaxial tension and 200,000 under biaxial tension. The computationally expensive FDEM simulations include 90,000 split evenly among PBX, shale, and tungsten under uniaxial loading. All simulations begin with randomized initial fracture patterns. Each entry includes temporal data capturing fracture propagation dynamics. This comprehensive dataset is designed to support the development of foundational or surrogate machine learning approaches for predicting material failure. While no such models are introduced here, the dataset lays a robust foundation for advancing future research and innovation in these areas.

## Background & summary

Fracturing phenomena are ubiquitous across numerous scientific and engineering domains and play a critical role in various areas such as subsurface geology especially subsurface flow in fractured reservoir^1,2^, structural engineering^3^, earthquake rupture dynamics and anthropogenic seismic hazards^4,5^, infrastructure integrity^6^, response of human-made systems to impulsive loads^7^, material design^8^, high explosives performance^9^, biomaterials such as bones^10^. Accurately modeling how fractures initiate, propagate, and interact with materials under stress is critical for advancing knowledge and developing resilient, safe, and efficient materials and structures. Despite significant advancements in both theory and computational techniques, simulating fracturing evolution under internal stress or external loading remains computationally intensive and inherently challenging due to the complexity of physical interactions and material heterogeneity, sometimes involving multi-physical fields.

Recent breakthroughs in machine learning (ML), particularly within natural language processing, have demonstrated the transformative potential of large-scale, data-driven models^11–13^. Scientific disciplines, however, face unique challenges in applying such models due to the high cost of data generation, quality control complexities, and the multi-modal nature of scientific datasets. Nonetheless, fields such as protein folding^14^, drug discovery^15^, climate science^16,17^, and computational chemistry^18,19^ have successfully leveraged large-scale ML, highlighting the potential for foundation models to overcome limitations of traditional simulation approaches.

In this work, we do not develop ML models, but rather introduce and release a comprehensive, high-quality dataset that captures the complexity of fracture evolution across materials and conditions. Our goal is to enable and accelerate research toward generalizable, large-scale ML models capable of inferring fracture patterns at the moment of material failure, and to support a broad range of scientific applications.

The dataset must include a sufficiently large number of diverse, random initial fracture patterns, as well as a variety of materials. Unfortunately, generating such a fracturing dataset through experiments is highly impractical. Preparing hundreds of thousands of material samples with different random initial fracture patterns is extremely difficult, if not impossible, and even if it were feasible, conducting that many experiments would be prohibitively time-consuming. To the best of our knowledge, no existing dataset in the domain of fracture and material failure provides images of both the random initial fracture patterns and their corresponding fracture patterns at the point of material failure under various boundary conditions.

**Fig. 1:**
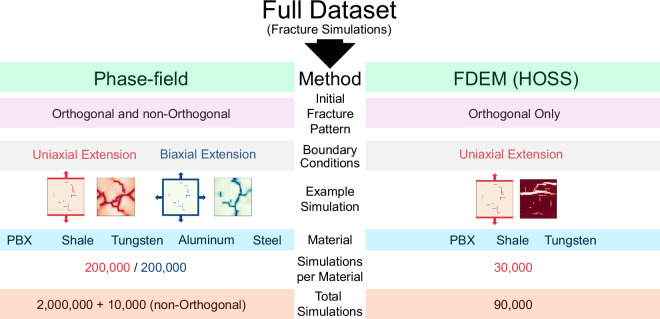
The data is divided into two distinct approaches – phase-field and FDEM (i.e. HOSS). Phase-field is composed of both uniaxial and biaxial extension boundary conditions while FDEM only includes uniaxial extension. The phase-field dataset includes five materials (PBX, shale, tungsten, aluminum and steel), while the FDEM dataset covers three materials (PBX, shale and tungsten). A small subset of data was made with randomly oriented initial fractures for the phase-field data (10,000) resulting from 1000 fractures  × the 2 boundary conditions  × the 5 materials. A full table of the material properties are presented in Table 1.

We leverage computational fracture mechanics and fracturing simulation to present a dataset that will provide the basis to support the training of multi-modal foundational models for predicting material failure. The dataset comprises simulations from two distinct computational approaches that incrementally capture greater physical fidelity as shown in Fig. 1: (1) a fully dynamic phase-field fracture method that approximates fracture propagation as a nonlinear diffusion process of a scalar phase-field variable; and (2) a fully dynamic, combined finite-discrete element method (FDEM) that explicitly simulates fracture initiation, propagation, and arrest under realistic loading conditions. Elements separate along predefined interfaces once failure criteria are met.

Comparatively, the two methods differ in their treatment of discontinuities, computational efficiency, physical fidelity, and fracture resolution. Phase-field simulations are computationally more efficient and suited for large-scale representation. In contrast, the high-resolution modeling of failure mechanics from FDEM are offset by a higher computational cost. In unison, they create a rich multi-resolution dataset that encompasses a comprehensive and complementary acuity for material failure.

This dataset not only spans a wide spectrum of physical complexity but also encompasses diverse data representations, including Cartesian grids and unstructured meshes, thus enabling versatile multi-modal model training. It supports various predictive tasks essential to fracture analysis, such as forecasting fracture patterns and time-to-failure predictions for different materials under varied loading conditions. Additionally, the dataset contains temporal data capturing fracture propagation over time. The dataset provides a robust foundation for exploring how large-scale models can generalize fracture predictions across multiple scales, materials, and loading scenarios, thus opening pathways for broader scientific impact beyond traditional, scenario-specific modeling approaches. By making the curated, multi-modal dataset publicly available, we aim to support broader efforts in data-driven modeling of fracture processes and material degradation. A non-exhaustive list of specific use cases of the dataset:

1. Training ML models to predict fracture evolution: The dataset includes temporal data capturing fracture propagation over time across multiple materials, enabling supervised learning for fracture growth prediction under extensional loading conditions.
2. Studying material-specific fracture behavior: With simulations spanning both brittle (e.g., PBX, shale) and ductile (e.g., tungsten, steel, and aluminum) materials, the dataset allows for systematic comparisons of failure mechanisms across material classes.
3. Investigating the influence of pre-existing fractures: The randomized initial fracture configurations enable studies of how fracture orientation, density, and connectivity affect the resulting failure patterns.
4. Developing surrogate models for high-throughput materials screening: The dataset provides a foundation for reduced-order models that can approximate fracture outcomes rapidly, which is valuable in materials design and optimization contexts.
5. Evaluating time-to-failure prediction algorithms: Temporal simulation data can support development of models that estimate when material failure occurs based on evolving internal stress or fracture states.

The rest of the paper is organized as follows: In Section 2, we briefly describe the two numerical methods for generating the fracturing simulation datasets as well as the method for generating the random initial fracture patterns. In Section 3, we describe the mechanical and fracture properties of the used materials, as well as display example fracturing simulation results generated by the numerical solvers. In Section 4, we discuss the data records. Then in Section 5, details on the technical validation are provided. In Section 6, we give information on how to use the dataset, including instructions on how to download and manipulate the dataset records. Section 7 describes code availability.

## Methodology

We use two different fracture physics models to generate the fracturing simulation dataset: phase-field and FDEM methods. The fundamental physics assumptions, fidelity, as well as numerical flexibility of these two models differ from one another, but complement each other, enriching the fracturing simulation dataset.

**Fig. 2:**
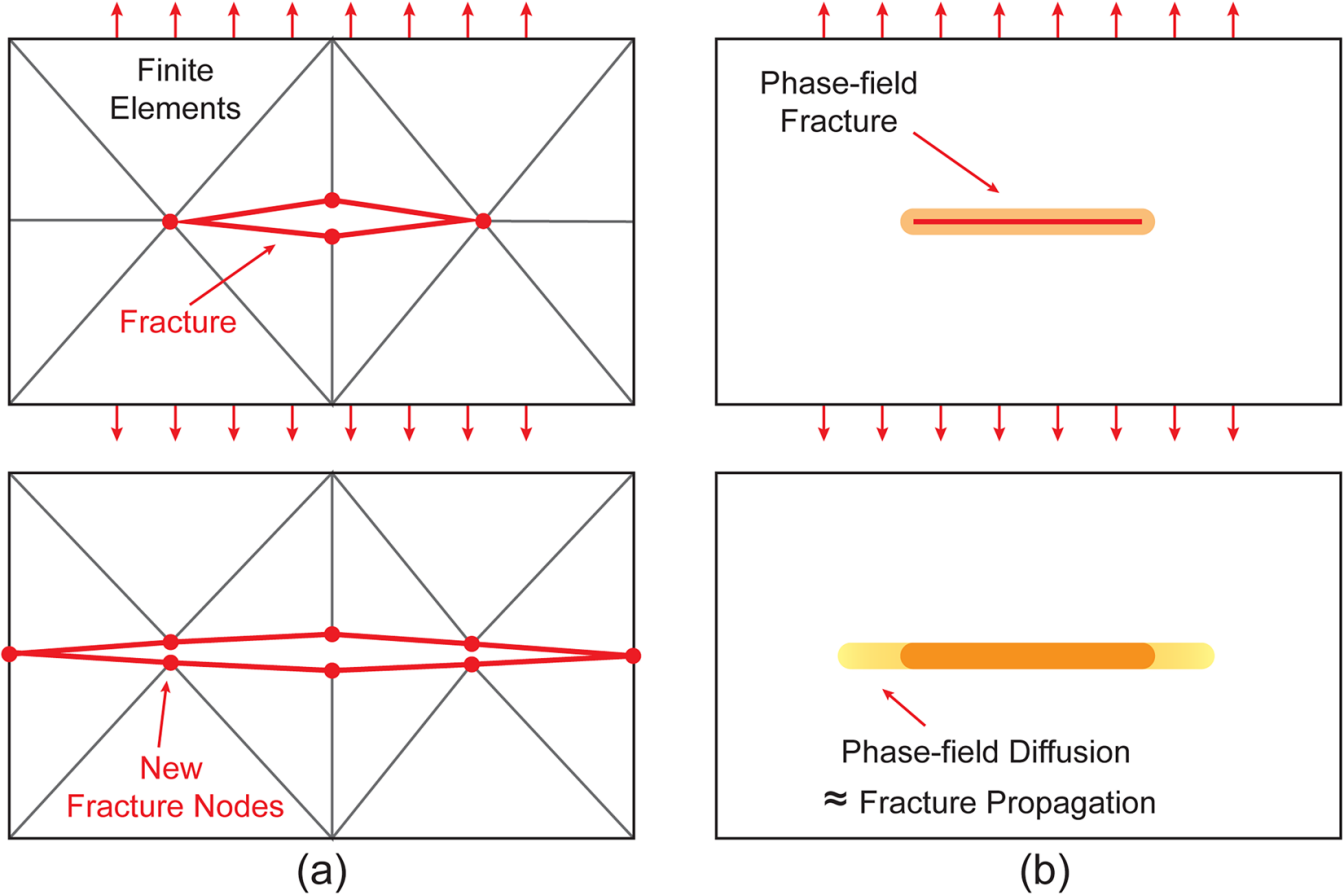
A schematic plot comparing the two fracturing physics models used in this study. The FDEM method (**a**) shows the fracture as a discrete discontinuous displacement between elements that has the ability to propagate based on mode I (shown here) and mode II behavior. It captures cohesive contacts between elements with a much higher fidelity and requires explicit stepping of the fracture evolution. In the phase-field approach (**b**) fractures are represented as a smooth, diffuse damage zone, where the material gradually transitions from intact to fully dislocated constrained by characteristic width 2*w*_0_. The continuous formulation allows for implicit solutions to the fracture propagation.

Figure 2 displays a schematic comparing the fracture patterns in the simplest possible case for the two fracturing physics models used in this study (horizontal fracture propagation due to uniaxial extension). In the FDEM approach, fractures are modeled as discrete separations between elements that evolve explicitly under mode I and II loading. In contrast, the phase-field method treats fractures as diffuse damage zones that transition smoothly from intact to failed material over a characteristic width 2*w*_0_, enabling implicit fracture propagation. In the following, we briefly review the core methodologies of these two physics models.

### Phase-field method for fracturing simulation

The phase-field method for fracture simulation approximates the evolution of material fractures with a nonlinear diffusion process of a continuous phase-field^20–22^. The most notable advantage of the phase-field method is its flexibility in simulating complex fracture propagation without the need of predefining or adaptively refining complex unstructured meshes along fracture propagation paths, which are essentially unknown beforehand.

In the phase-field fracture propagation theory, the elastic constitutive relation of the background material reads^22,23^:

1 $$\sigma ({\bf{u}})=\left[(1-\eta ){(1-\psi )}^{2}+\xi \right]{\bf{C}}\,:\varepsilon ,$$

where *σ* is the stress tensor, **u** is displacement, **C** = **C**(**x**) = *C*_*ijkl*_(**x**) is the fourth-order elasticity tensor of the matrix with up to 21 independent components; $\varepsilon =\frac{1}{2}\left(\nabla {\bf{u}}+\nabla {{\bf{u}}}^{{\rm{T}}}\right)$ is the strain tensor; *ξ* is used to prevent stiffness from reaching 0 during full fracture, *η* is a small positive number to avoid instability at the fracture *ψ*. The Neumann boundary condition for *σ* is *σ* ⋅ **n** = **t**_*N*_, where **n** is the normal vector of the boundary ∂*Ω*_*N*_, *Ω* is the computational domain, and **t** is a traction vector.

In this work, we simulate dynamic rather than quasi-static fracture propagation to better capture the fracture evolution under dynamic boundary conditions (e.g., constant-velocity tension forces applied at boundaries). Thus, rather than solving the static elastic deformation equation in Eq. (1), we consider the dynamic elastic constitutive relation in a degraded medium:

2 $$\rho {\partial }_{t}^{2}{\bf{u}}=\nabla \cdot \sigma +{\bf{f}},$$

where *σ* follows Eq. (1), **f** = **f**(**x**, *t*) is the dynamic external force term, and *ρ* is the mass density of the background medium. This is effectively the elastic-wave propagation equation in a degraded medium. The evolution of the fracture phase-field *ψ* is driven by the so-called positive elastic strain energy. Based on the principle of variation, the governing equation for the fracture evolution can be written as^22,23^:

3 $${G}_{c}\left({w}_{0}^{-1}\psi -{w}_{0}{\nabla }^{2}\psi \right)=2(1-\psi ){H}^{+}(\varepsilon ,t),$$

where 2*w*_0_ is the characteristic width of a fracture and *G*_*c*_ is the critical energy release rate; *H*^+^(*ε*, *t*) is a history energy field defined as the maximum positive elastic energy at **x** up to *t*:

4 $${H}^{+}(\varepsilon ,t)=\mathop{\max }\limits_{\tau \in [0,t]}{\Phi }^{+}(\varepsilon ,\tau ).$$

While there are a number of choices to define the positive elastic strain energy function *Φ*^+^, here we choose one based on the elastic tensor **C** in a general form for computing the following elastic energy:

5 $${\Phi }^{+}(\varepsilon ,t)=\frac{1}{2}{({\varepsilon }^{+})}^{{\rm{T}}}\,{\bf{C}}\,{\varepsilon }^{+},$$

where the tensile-positive elastic strain tensor reads

6 $${\varepsilon }^{+}={\left[{\varepsilon }_{xx}^{+},{\varepsilon }_{yy}^{+},{\varepsilon }_{zz}^{+},2{\varepsilon }_{yz},2{\varepsilon }_{xz},2{\varepsilon }_{xy}\right]}^{{\rm{T}}},$$

and the tensile-positive strain tensor being

7 $${\varepsilon }_{ij}^{+}=\frac{1}{2}\left({\varepsilon }_{ij}+| {\varepsilon }_{ij}| \right),$$

with ${\varepsilon }_{ij}=\frac{1}{2}({\partial }_{i}{u}_{j}+{\partial }_{j}{u}_{i})$ being the strain tensor. The above definition allows us to define the history energy function for both isotropic and anisotropic materials in a unified formulation. The resulting coupled dynamic fracture evolution system reads:

8 $$\rho {\partial }_{t}^{2}{\bf{u}}-\nabla \cdot \sigma ={\bf{f}},$$

9 $$\sigma ({\bf{u}})=[(1-\eta ){(1-\psi )}^{2}+\eta ]{\bf{C}}\,\varepsilon ,$$

10 $$\varepsilon =\frac{1}{2}(\nabla {\bf{u}}+\nabla {{\bf{u}}}^{{\rm{T}}}),$$

11 $${G}_{c}\left({w}_{0}^{-1}\psi -{w}_{0}{\nabla }^{2}\psi \right)=2(1-\psi ){H}^{+}(\varepsilon ,t).$$

This system of coupled partial differential equations requires solving two partial differential equations: one is the linear elastic wave equation in a degraded medium, and the other is the nonlinear phase-field diffusion equation. We solve them using the lowest-order spectral-element method and Newmark-beta implicit scheme. To capture fracture propagation in ductile, elastoplastic materials such as metals (e.g., tungsten, aluminum, steel, etc.), we incorporate the von Mises yield criterion^24^ into the constitutive relation of the background medium. This involves modifying the effective elastic tensor to reflect the reduction in stiffness associated with plastic yielding and accumulated damage.

### Combined finite discrete-element method for fracturing simulation

Researchers at Los Alamos National Laboratory have developed the Hybrid Optimization Software Suite (HOSS)^25^, based on the FDEM, to simulate complex mechanical behaviors of solid materials, including fracturing and fragmentation^26^. HOSS is widely used due to its robustness, advanced algorithms, massive parallelization features, and versatile capabilities. HOSS results have been validated by numerous independent experiments^25^. The non-exhaustive list of HOSS applications include the following: experimental rock mechanics (e.g. triaxial uniaxial), well-bore and drilling stability, building integrity, seismic studies, high velocity impacts, thermal-hydraulic-mechanic behavior, high explosive performance, tissue and cell experiments, and weapons penetration^25,27– 32^.

For material fracturing, the discrete system that solves the coupled elastic/ductile deformation and fracture propagation can be written as

12 $${\bf{M}}\frac{{d}^{2}{\bf{x}}}{d{t}^{2}}+{\bf{C}}\frac{d{\bf{x}}}{dt}={\bf{F}},$$

where **M** is the mass matrix, **C** is the damping matrix, **F** is the equivalent nodal force, including all forces distributed to hte node, such as body forces, forces due to material deformation, contact forces between solid bodies, and cohesion forces in damage^26,27,33,34^; **x** is the vector enclosing the node displacement field^35^.

In this study the numerical domain was discretized using triangular finite elements, with their rotaiton, deformation, and stress evaluation through a multiplicative decomposition framework^29,35^. To identify contact interactions between elements, a contact detection algorithm with linear computational and memory complexity was employed^36^. A potential field-based penalty method was applied to handle the frictional contact interaction in the contact zones^37^. An explicit central difference time integration scheme was used to obtain the temporal evolution of the system^38^.

In this work, a generalized traction-separation model is used to simulation the damage in the material. Specifically, the traction is defined from the gradient of the interfacial potential which is a funciton of the components of the separation vector^27^. In a simplified form, the traction is defined as:

13 $${\boldsymbol{p}}=({f}_{t}{\boldsymbol{n}}+{f}_{s}{\boldsymbol{t}})z(d)$$

where *f*_*t*_ and *f*_*s*_ are the tensile and shear strengths, while ***n*** and ***t*** are the normal and tangential directions. The function *z*(*d*) characterizes the shape of the traction-separation curve and is expressed as a function of the damage variable *d*. Damage variable *d* takes a value between 0 and 1.0 and is given by:

14 $$d=\sqrt{{d}_{n}^{2}+{d}_{t}^{2}}$$

where

15 $${d}_{n}=\frac{{\delta }_{n}}{{\delta }_{nc}};{d}_{t}=\frac{{\delta }_{t}}{{\delta }_{tc}}$$

are the normal and tangential damage components. The *δ*_*n*_ and *δ*_*t*_ are the normal and tangential components of the separation while *δ*_*nc*_ and *δ*_*tc*_ are the maximum tensile and shear separations.

The fracture release energies in mode I and mode II are given by:

16 $${G}_{I}={f}_{t}{\delta }_{nc}{\int }_{0}^{1}z(d)$$

17 $${G}_{II}={f}_{s}{\delta }_{tc}{\int }_{0}^{1}z(d)$$

Note that damage $d=\frac{\delta }{{\delta }_{c}}\le 1.0$ will evolve from *δ* → *δ*_*c*_ where *δ*_*c*_ is a critical value at which *f*(*δ*_*c*_) = 0 whereby the cohesive resistance vanishes leading to complete decohesion and possible fragmentation, capturing crack propagation and the transition from continuous to fully discrete material behavior.

In addition, we employ in-house state-of-the-art discretized contact solutions to handle contact detection and contact interaction^25,28^. The contact detection follows the the MRCK scheme^39,40^ where the physical space (two-dimensional here) is uniformly partitioned into square cells of size *h*. Each target sample (points along the element boundaries) is mapped to a cell (*i*, *j*) with $i=\frac{x-{x}_{0}}{h}$ and $j=\frac{y-{y}_{0}}{h}$ and stored in a single MR-linear sort reduced to determine whether the contactor and the target share at least one square cell. In the two-dimensional implementation here, a candidate pair is accepted if a target point lies within the contactor triangle. The triangle-to-point contact interaction approach is used such that target triangles are discretized into boundary points that act as Gauss integration points for evaluation distributed contact forces. The MRCK contact search algorithm identifies contact pairs between contactor triangles and target points, with interaction computed only when a target point lies within a contactor triangle.

Material failure analysis requires careful consideration of key indicators to accurately determine the point of failure. In addition, the data must be processed in a way that captures critical trends while maintaining consistency between the initial setup and the final fracture domains. Therefore, two important data processing steps must take place to ensure that there is (1) a systematic choice of the time-to-failure, and (2) a consistent data structure between the initial and final time steps. To resolve these difficulties, simulation results are post-processed by incorporating some of the unique variables tracked by the HOSS simulations which are not necessarily important to the final fracture results. HOSS tracks the entire solid kinetic energy of the system and also the stress tensor for each element. To determine the time-to-failure we use an automated picking routine that follows the following steps: (1) evaluate the total simulation stress and total kinetic energy for each time step, (2) identify all peaks in the stress and kinetic energy throughout the simulation, (3) find the “key” kinetic energy peak with the steepest drop, which corresponds to failure onset, (4) find the stress peak that first occurs after the “key” kinetic energy peak, which marks the transition where the kinetic energy spikes may indicate failure events, (5) determine the final failure time step by computing the gradient of the stress data and picking where the stress stops decreasing after previously being negative, which represents the moment when the material stops significantly responding to stress likely indicating failure. In this way, each simulation incorporates a systematic choice for the time-to-failure. Through visual inspection we found this method to produce the most consistent times where fractures had nearly or fully propagated across the samples. Material failure can take various forms, but these results provide a conservative estimate of the time-to-failure. In reality, failure may occur earlier if other factors, such as sudden stress drops, are considered.

Since the simulations produce displacement between fractures the initialized domain increases in size vertically as shown in Fig. 9. These results produce an unstructured grid of information that may also be long past the first time-to-failure step. To resolve these difficulties results are post-processed using the PyVista package^41^. HOSS retains unique global nodal identification numbers (IDs) which for our triangular mesh means we have unique edges for the entire simulation. Each edge retains information called “damage” that represents the loss of cohesion between its edge and neighboring triangle edge^27^. It is important to note that within each simulation of the dataset each edge will retain a value between 0.0 and 1.0, but only values equal to 1.0 should be classified as a true fracture. After selecting the time step at which failure occurs in the simulation, we map the damage values from the final and intermediate steps back onto the initialized mesh. This process preserves both the edges of elements that fractured at the earliest failure time and the original grid structure of the domain. We evaluate three of the most unique material end members similarly explored in the phase-field model (i.e. PBX, shale, and tungsten).

### Generation of random initial fracture patterns

Each fracture simulation begins with a randomly generated initial fracture pattern, which serves as the starting point for subsequent fracture propagation. Our first distribution of data consists of orthogonally aligned initial fracture patterns randomly arranged in a 2D computational domain with a size of 0.25 m in each dimension. In 2D, the key properties of the initial fracture patterns are: location, length, and orientation. In order to generate a sufficiently large dataset that ensures random fracture properties, we adopt a procedure as described below.

First, we draw three sets of values from three distinct uniform distributions:

18 $$({S}_{x},{S}_{z},L)\in \left[\,{{\mathcal{U}}}_{\xi }({\delta }_{x},{H}_{x}-{\delta }_{x}),\,{{\mathcal{U}}}_{\zeta }({\delta }_{z},{H}_{z}-{\delta }_{z}),\,{{\mathcal{U}}}_{\eta }({L}_{\min },{L}_{\max })\right],$$

where *S*_*x*_ and *S*_*z*_ represent the starting coordinates of a fracture in x- and y-dimensions, *L* is the length of a fracture, ${\mathcal{U}}(a,b)$ is a uniform random distribution with a lower bound of *a* and an upper bound of *b*, with *ξ*, *ζ* and *η* being three different random realization seeds. In addition, ${L}_{\min }=0.01$ m and ${L}_{\max }=0.05$ m represent the minimum and maximum lengths of a fracture, respectively, *H*_*x*_ = *H*_*z*_ = 0.25 m is the domain size, and *δ*_*x*_ = *δ*_*z*_ = 0.02 m represent the “buffer” zone of the fractures to avoid having fractures on the boundaries. The coordinates of the termination point for each fracture are calculated relative to the starting coordinates and orientation:

19 $$({E}_{x},{E}_{z})=({S}_{x},{S}_{z})+(L\,\cos (\theta ),L\,\sin (\theta )),$$

where *θ* is the polar orientation of a fracture. By setting *θ* = 0 or *π*/2, we can get a horizontal or vertical fracture. Furthermore, in each initial fracture pattern the number of horizontal/vertical fractures is also random:

20 $$({N}_{h},{N}_{v})\in [{{\mathcal{U}}}_{h}({N}_{\min },{N}_{\max }),\,{{\mathcal{U}}}_{v}({N}_{\min },{N}_{\max })],$$

with ${N}_{\min }=3$ and ${N}_{\max }=15$ representing the minimum and maximum numbers of fractures in a model, respectively. The total number of fractures for different fracture patterns therefore satisfies

21 $$2{N}_{\min }\le {N}_{f}\le 2{N}_{\max }.$$

**Fig. 3:**
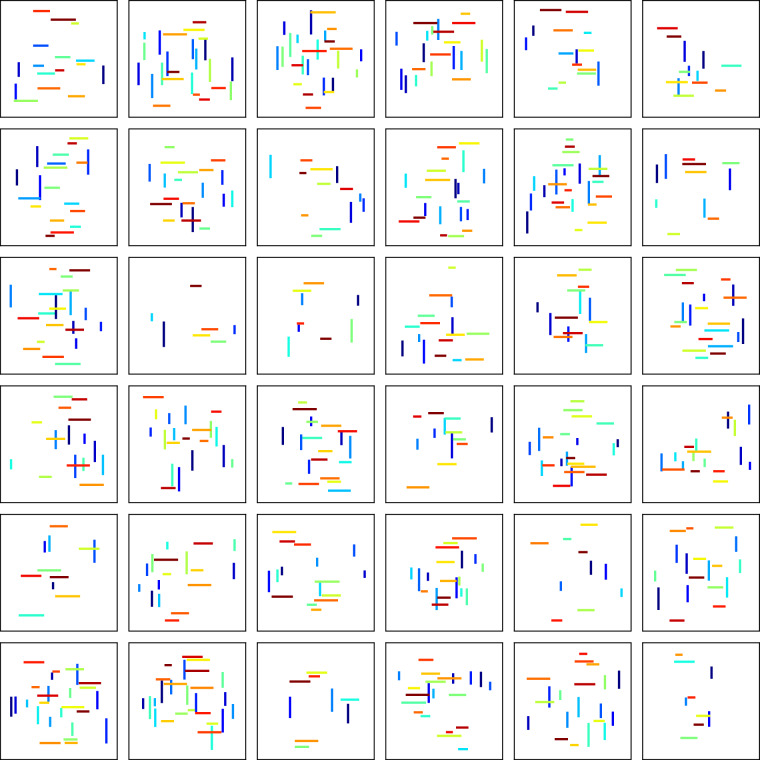
Examples of randomly generated initial fracture patterns with orthogonal orientations used in fracturing simulations. Colors in the plots represent distinct fractures. The size of the simulated domain is *H*_*x*_ = *H*_*z*_ = 0.25 m (omitted for brevity).

In this way, not only the fracture locations vary, but also the number of horizontal/vertical fractures. Using this method, we generated a total of 200,000 orthogonal initial fracture patterns. Figure 3 displays 36 examples, where in each fracture pattern, fractures are colored with different colors for better visual distinction. These 200,000 initial fracture patterns are included in our open-access dataset.

To support future validation and generalization of ML models on unseen fracture samples, we generate 1,000 random non-orthogonal fracture patterns where the fractures are not necessarily orthogonal with one another. In this case, each model contains a single fracture set, and fracture orientations are randomly assigned by drawing from a uniform distribution:

22 $$\theta \in {{\mathcal{U}}}_{\theta }\left(0,\frac{\pi }{2}\right).$$

**Fig. 4:**
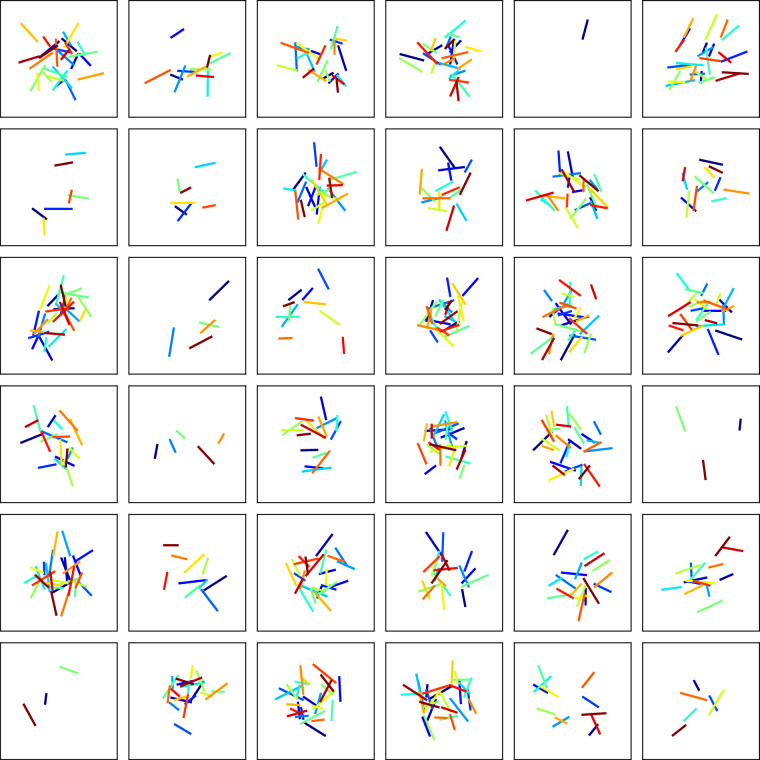
Examples of randomly generated initial fracture patterns with non-orthogonal orientations used in fracturing simulations. Colors in the plots represent distinct fractures. The size of the simulated domain is *H*_*x*_ = *H*_*z*_ = 0.25 m (omitted for brevity).

We display 36 examples of the non-orthogonal fracture patterns in Fig. 4.

### Fracture Simulation Results

In the following subsections, we describe the properties of the materials that we used to create the datasets and highlight the material dependent differences in fracture propagation. We also show a handful of examples of the simulated fracture patterns using the phase-field and HOSS simulators.

#### Material properties

We perform fracturing simulations for five materials: PBX, shale rock, tungsten, steel, and aluminum. PBX and shale are simulated as elastic materials, while the other three as ductile elastoplastic materials.

The PBX is representative of a brittle elastic fracture behavior common to many geo-materials like rocks and concrete. This material exhibits brittle behavior, with some localized damage before a full fracture which, at the strain rates applied by our boundary conditions, implies dynamic failure.

The shale is representative of a brittle elastic failure behavior that incorporates anisotropy – a key characteristic in geomechanics and reservoir engineering. It is the only material which anisotropy is accounted for. The mechanically anisotropic bedding planes produced by the clay-rich shale causes resistance to fracture perpendicular to the bedding and lower resistance to fracture parallel to the bedding. This anisotropy allows for fractures to propagate slowly, and in greater abundance, over time before sudden failure. The elasticity of the shale is specified with elasticity constants in the Voigt notation (i.e., *C*_*ij*_) rather than its original form *c*_*ijkl*_. Since, we only simulate fracturing in a 2D plane (*x* − *z* plane or equivalently *x*_1_ − *x*_3_ plane), we only display the relevant elasticity parameters on this plane (i.e., *C*_11_, *C*_13_, *C*_33_, and *C*_55_).

Tungsten is a high density metal that is representative of plastic deformation behavior important to many industrial alloys and other metals. For the phase-field-based fracturing simulations, we set the Griffith critical energy release rate (*G*_*c*_) of tungsten to 500 J/m^2^, which is a slightly higher than usual value for tungsten at room temperature. For FDEM we use both modes: mode I fracture energy (*G*_*I*_), and mode II (*G*_*II*_) fracture energy. We simulated fracturing for tungsten at elevated temperatures.

The aluminum we use here is a type of hardened, high-strength aluminum alloy rather than pure aluminum. Therefore, its Poisson’s ratio (0.25) is notably smaller than that of the pure aluminum (typically 0.31 to 0.34). Henceforth, we continue to use the term “aluminum” to represent this aluminum alloy for brevity.

We summarize the elasticity, ductility, and fracture-related parameters of the five materials in Table 1.

**Table 1:** Properties for the five materials for fracturing simulation.

Property	PBX	Shale	Tungsten	Aluminum	Steel
Density (kg/m^3^)	1.82 × 10^3^	2.075 × 10^3^	19.25 × 10^3^	2.7 × 10^3^	7.85 × 10^3^
Young’s Modulus (Pa)	1.0 × 10^10^	—	4.0 × 10^11^	6.49 × 10^10^	2 × 10^11^
Poisson’s Ratio	0.36	—	0.28	0.25	0.3
C_11_ (Pa)	—	3.125738 × 10^10^	—	—	—
C_13_ (Pa)	—	3.399087 × 10^9^	—	—	—
C_33_ (Pa)	—	2.248732 × 10^10^	—	—	—
C_55_ (Pa)	—	6.486048 × 10^9^	—	—	—
Yield Strength (Pa)	—	—	7.5 × 10^8^	2.5 × 10^8^	6 × 10^8^
Hardening Modulus (Pa)	—	—	5.0 × 10^9^	2.5 × 10^8^	2.5 × 10^9^
^†^Critical Energy Release Rate (J/m^2^)	640.885	50	500	1 × 10^4^	2.5 × 10^5^
^†^Characteristic Width (m)	0.004	0.002	0.002	0.004	0.004
*Coulomb Friction	0.6	0.6	0.6	—	—
*Tensile Strength (Pa)	0.5 × 10^6^	1.0 × 10^7^	8.5 × 10^8^	—	—
*Shear Strength (Pa)	2.0 × 10^6^	4.0 × 10^7^	4.905 × 10^8^	—	—
*G_*I*_ (J/m^2^)	125	100	12750	—	—
*G_*II*_ (J/m^2^)	500	400	7358	—	—
*Characteristic Width (m)	0.003	0.003	0.003	—	—

Properties marked with ^†^are exclusive to phase-field simulations and those marked with *are exclusive to HOSS.

#### Phase-field-based fracturing simulation results

With the generated random fracture patterns and the mechanical properties of the five materials showed above, we performed 200,000 fracturing simulations using our in-house solver. We discretize the random fracture patterns using 127 × 127 regular first-order spectral elements^42^, resulting in a total of 128 × 128 degrees of freedom for the phase-field equation, Eq. (3), and a total of 2 × 128 × 128 degrees of freedom for the elastic constitutive equation, Eq. (2) in the 2D case.

For each initial fracture pattern and material, we perform two simulations under different boundary conditions: In the first one we apply uniaxial extension (vertical tension force) with a constant velocity of 1 m/s at the top and and bottom boundaries (i.e., *v*_top_ = − 1 m/s, *v*_bottom_ = 1 ms, assuming that the *z*-axis points from the bottom to the top); in the second one we apply biaxial extension on all the boundaries of a sample (i.e., *v*_top_ = *v*_left_ = − 1 m/s, *v*_bottom_ = *v*_right_ = 1 m/s, assuming that the *x*-axis points from the left to the right).

**Fig. 5:**
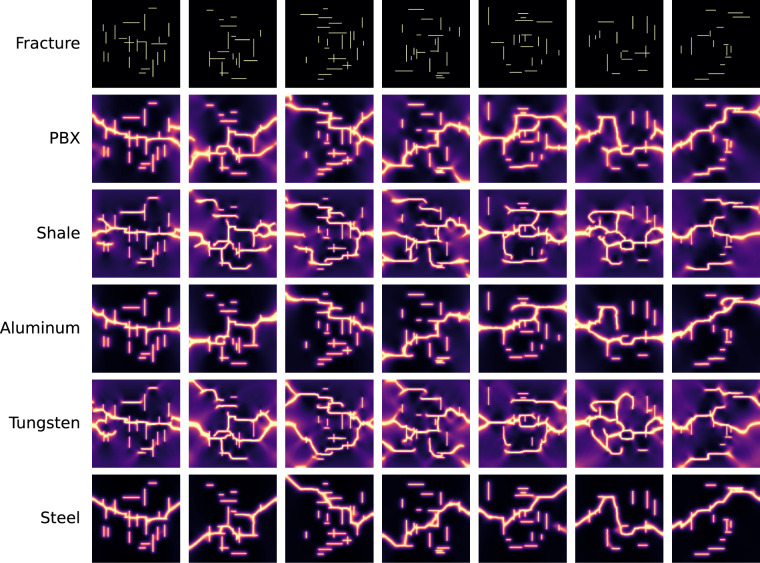
Examples of phase-field simulations for five materials with randomly generated orthogonal fractures subjected to uniaxial boundary conditions.

Figure 5 displays seven examples of fracturing simulations under uniaxial extension for each of the five materials. The top row shows the randomly generated initial fracture patterns, while each subsequent row illustrates the final fracture failure patterns for a specific material based on those initial conditions. The rows show how the mechanical properties of different materials influence their failure behavior. The columns emphasize that, for a given material, the initial fracture geometry strongly dictates the final fracture outcome at the point of failure.

**Fig. 6:**
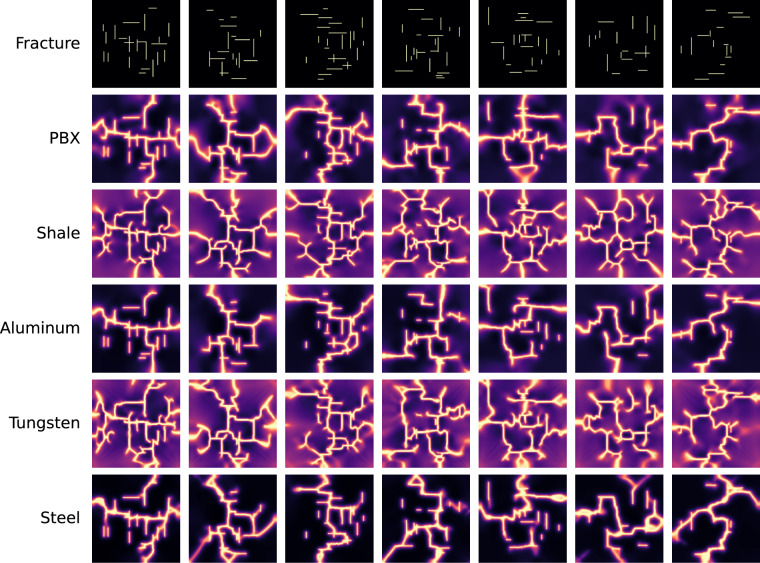
Examples of phase-field simulations for five materials with randomly generated orthogonal fractures subjected to biaxial boundary conditions.

By comparison, Fig. 6 shows seven examples of fracturing simulation under biaxial extension for the five materials. In this case, we observe fractures growing in both directions (vertical and horizontal), resulting in more complex fracture patterns than the ones observed for the uniaxial boundary conditions. Under vertical tension (uniaxial), fractures mostly grow horizontally or diagonally, while fractures growing vertically are rare. Since, fracturing is a complex mechanical process that involves multiple closely coupled factors, it is challenging to isolate the sole factor that leads to some of the failure pattern similarities between the different materials.

**Fig. 7:**
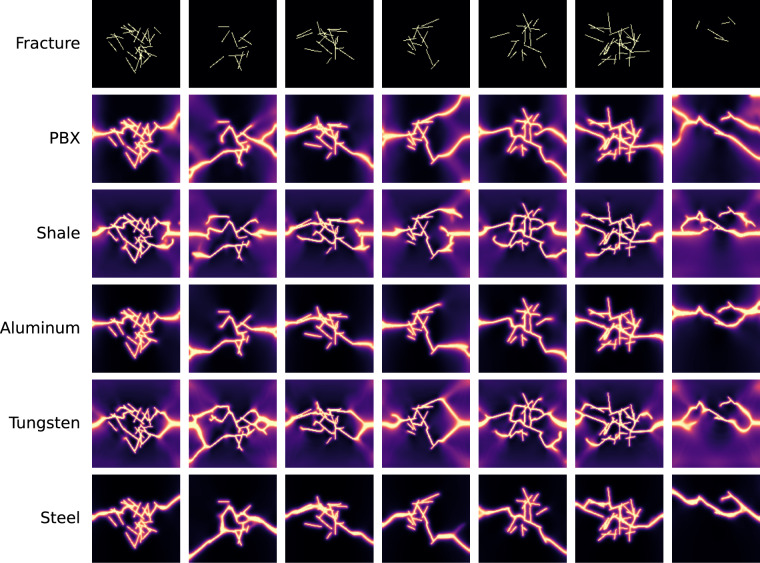
Examples of phase-field simulations for five materials with randomly generated non-orthogonal fractures under uniaxial boundary conditions.

**Fig. 8:**
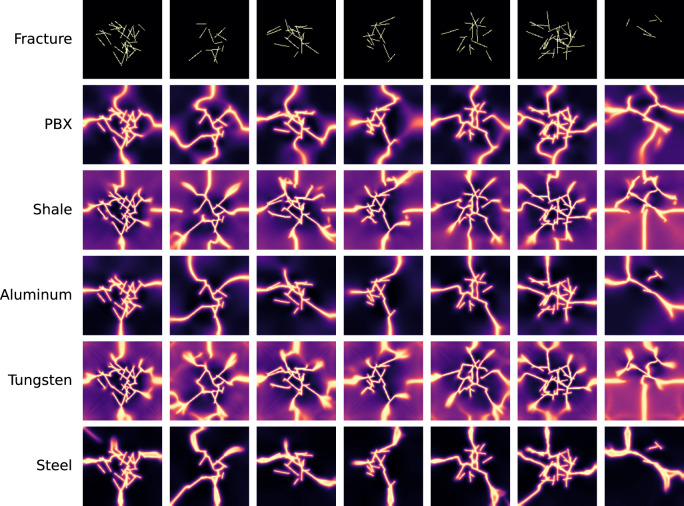
Examples of phase-field simulations for five materials with randomly generated non-orthogonal fractures under biaxial boundary conditions.

We also perform fracturing simulations for random non-orthogonal fracture patterns using uniaxial and biaxial boundary conditions. We display examples for non-orthogonal fractures in Fig. 7 with uniaxial extension and in Fig. 8 with biaxial extension. Because of the notable differences in the initial fracture patterns compared with those in Figs. 5,6, the final material failure fracture patterns also differ significantly. For pre-existing fracture patterns with low connectivity (e.g., the fourth and the seventh columns in Fig. 7), different materials exhibit notably different failure patterns.

#### HOSS fracturing simulation results

**Fig. 9:**
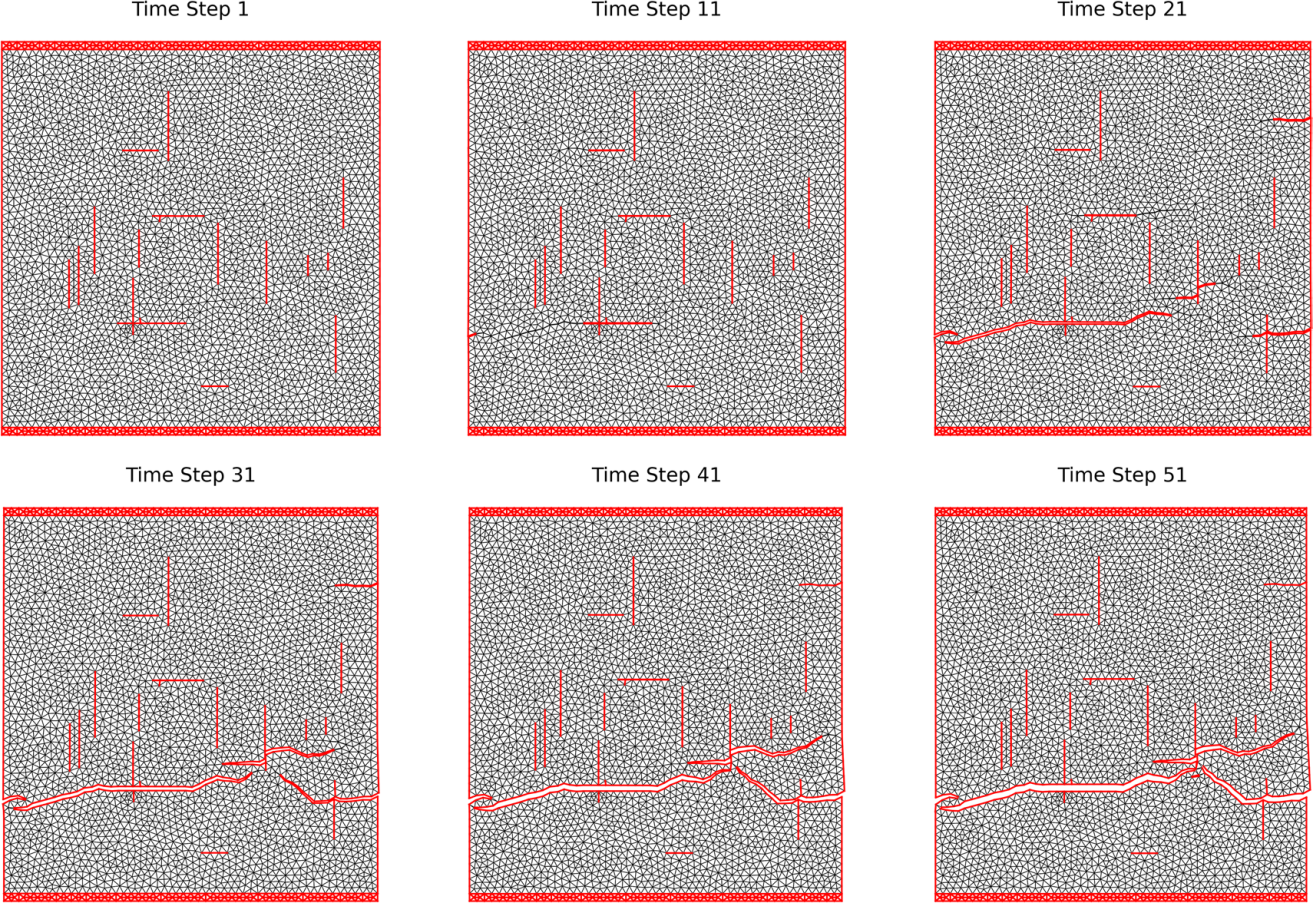
Single HOSS fracture example at different time steps for PBX. Note, time-to-failure could be earlier in the simulation but requires post-processing from the final fracture pattern.

For the HOSS fracturing simulations, we use the same set of random orthogonal initial fracture patterns generated for the phase-field method simulations, as shown in Fig. 3. Since, these simulations are much more expensive than the phase-field ones, we consider only three materials (PBX, shale and tungsten) and one type of boundary conditions (uniaxial extension). These three materials were chosen to cover the unique range of material types discussed in section 6- quasi-brittle (PBX), anisotropic brittle (shale), and plastic deformation (tungsten). To accurately impose the boundary conditions (tension), we confine the 0.25 m by 0.25 m domain by 5-mm plates the top and bottom, as indicated by Fig. 9. We assume free boundary conditions for the sides of the model and use a linear ramping (0 to 10^−4^ s) nodal velocity boundary conditions for the plates that are ±1 m/s (tension). The ramping allows the boundary conditions to slowly approach the ±1 m/s to avoid erroneous shearing at the boundaries of the material.

Different from the phase-field solver used in this work, HOSS is a solver that uses an explicit time stepping scheme during simulation limited by the critical time step *t*_*c*_ which is a function of the smallest element size *h* and wave speed *c* (*t*_*c*_ ∝ *h*/*c*). This condition ensures that waves do not propagate across more than one element per time step, maintaining numerical stability. To ensure numerical stability, we use a fixed time increment of 10^−8^ s. The mesh and element sizes are relatively fixed and do not vary during the simulation, such that our chosen critical time represents the largest stable time step that can be used throughout the analysis. As we cannot anticipate the exact time-to-failure of the material during the simulation, we allow each simulation to run for a specified wall-clock time of eight hours using eight MPI domains and CPU cores, which is the typical duration needed to achieve full failure.

Figure 9 displays an example of fracturing in PBX produced by our HOSS solver over six snapshots. It could be seen that the propagation of pre-existing fractures initiates from the lower-left part where two fractures intersect, and propagates horizontally over the sample until the sample fails. Although there are other fractures that propagate before failure, this initially propagated fracture in the lower half of the model dominates the fracturing process.

**Fig. 10:**
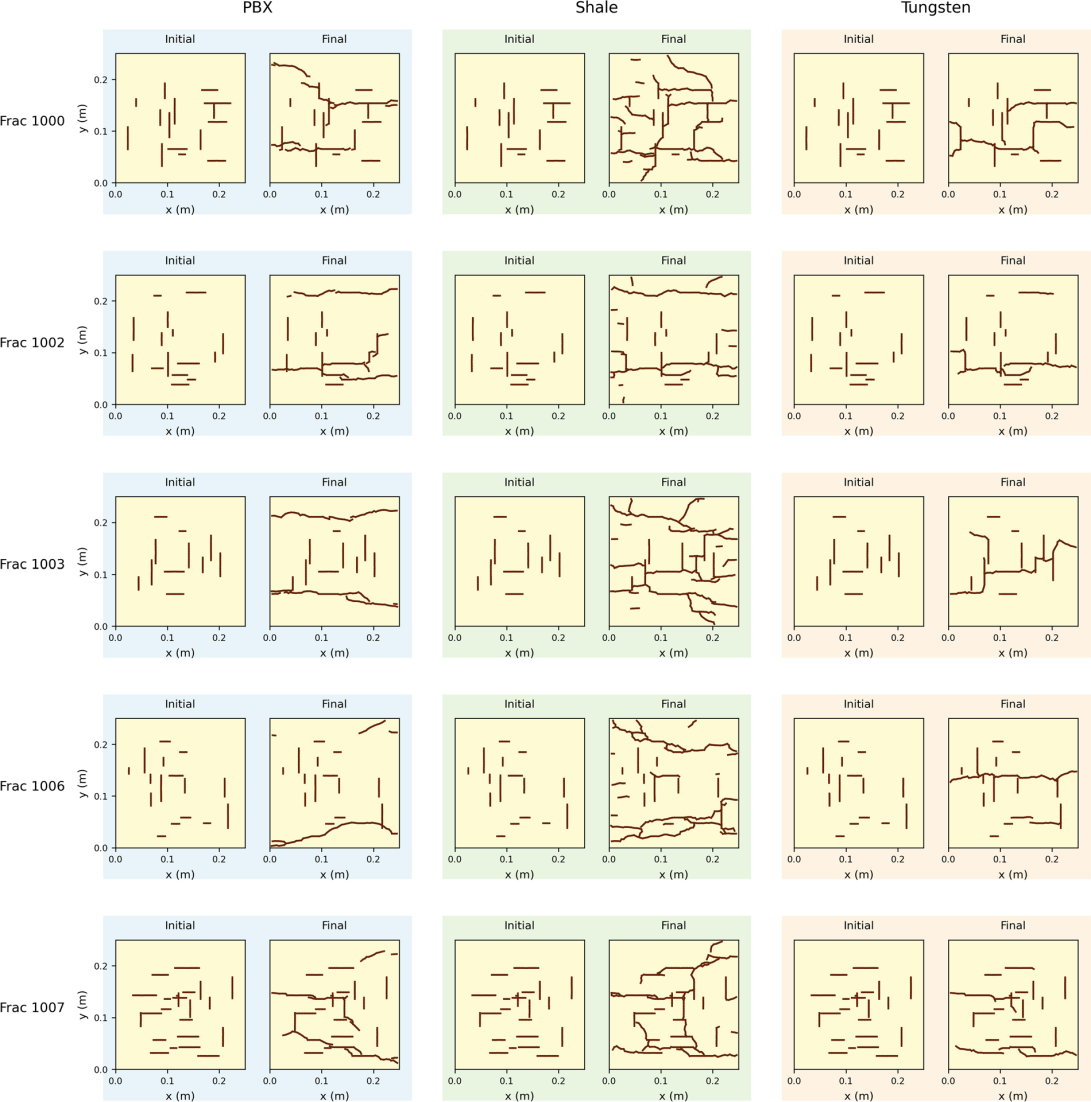
Multiple examples of fracturing simulations in HOSS for the three materials tested in that portion of the dataset with initial orthogonal fracture patterns and final fracture pattern at time of failure.

An example of the initialized fracture patterns and final results of the three materials PBX, shale, and tungsten is shown in Fig. 10.

## Data Records

The dataset supporting this study has been made available on Hugging Face Datasets https://huggingface.co/datasets/smartFRACs/material_fracturing^43^.

The full dataset is split into two primary folders: HOSS and PHASE-FIELD, corresponding to the two numerical solvers. Within each of these folders, data is further categorized by material type and boundary condition (phase-field only). The simulation outputs are stored as compressed .tar.gz archives, each containing approximately 1000 .h5 files representing individual fracture simulations. Additional metadata, file descriptions, and usage instructions are provided in the accompanying README file at the dataset URL.

## Technical Validation

To establish confidence in the numerical methods implemented here, we outline in the following subsections how each simulation approach was validated. To validate our implementation of the phase-field method – developed specifically for generating this dataset – we compare it against a set of benchmark crack-propagation problems. We ensure consistency and correctness of fracture evolution to common examples in the literature. For the FDEM simulations, which are resolved from the more mature and similarly validated HOSS software, we highlight common issues encountered during large-scale runs and the corrective measures applied to ensure result integrity.

### Phase-field method

Phase-field method has been widely used in the last decade for simulating crack and fracture propagation due to its flexibility and accuracy. Here, we perform three simulations in three samples of different sizes and material properties to verify the efficacy and accuracy of our phase-field solver.

**Fig. 11:**
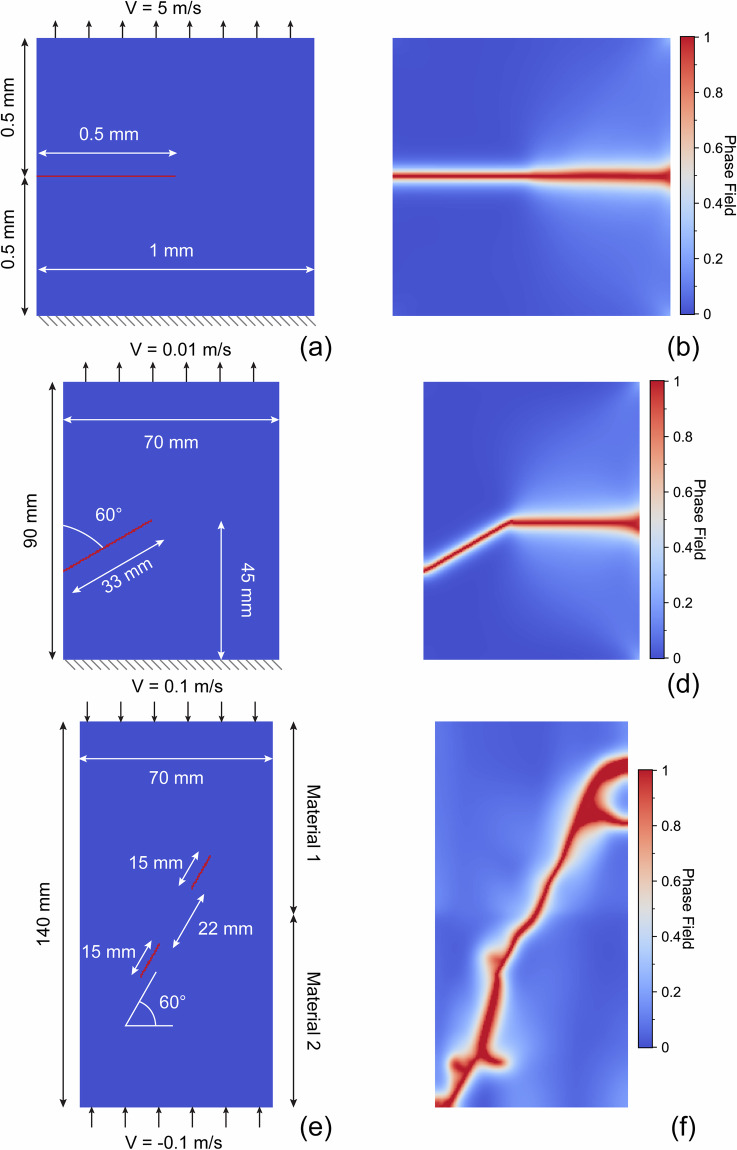
Three examples for verifying the efficacy and accuracy of our in-house phase-field solver. (**a**–**b**) Sample with a horizontal boundary crack. (**c**–**d**) Sample with a tilt boundary crack. (**e**–**f**) Sample with two tilt interior cracks. Panels (**a,** **c,** **e**) display the sample dimensions, crack positions, and boundary conditions while (**b,** **d,** **f**) display the simulation results and final fracture evolution using the phase-field solver. Result in panel (**b**) show a good match with Fig. 1c of^44^. Result in panel (**d**) show a good match with Fig. 4b of^22^. Result in (f) show a good match with Fig. 7j and k of^46^.

In the first example, we simulate the evolution of a boundary crack in a tiny, square sample under tensile boundary conditions. This test example aims to replicate the numerical experiment in^44^. Figure 11a displays the configuration of the experiment. The size of the sample is 1 mm in both dimensions. The sample is discretized with 200 × 200 regular rectangle spectral elements with a grid size of 5 × 10^−6^ m. The characteristic fracture width is *l*_0_ = 2 × 10^−5^ m. The sample has a Young’s modulus *E* of 210 GPa, a Poisson’s ratio *ν* of 0.3, a density *ρ* of 4,000 kg/m^3^, and a critical energy release rate *G*_*c*_ of 2,700 J/m^2^. A 0.5-mm long crack, extending from the left boundary to the horizontal center, is placed at the vertical center position. The bottom boundary of the sample is fixed. A velocity of 5 m/s is applied at the top boundary, generating an effective tensile boundary condition. Figure 11b shows the resulting fracture pattern at the failure time, which shows that the fracture horizontally propagates to the right boundary from the center along a straight line. The result is consistent with the simulation result displayed in Fig. 1c of^44^.

In the second example, we simulate fracture propagation using the phase-field solver in a larger, rectangle sample under tensile boundary conditions. This test example aims to replicate the numerical example in^22^. The sample, as displayed in Fig. 11c, is 90 mm in height and 70 mm in width. The sample is discretized with *N*_horizontal_ × *N*_vertical_ = 156 × 200 regular rectangle spectral elements with a grid size of 4.5 × 10^−4^ m. The characteristic fracture width is *l*_0_ = 15 × 10^−4^ m. The properties of the medium are *E* = 280 MPa, *ν* = 0.45, *ρ* = 1, 250 km/m^3^, and *G*_*c*_ = 27 J/m^2^, resembling CO poly (ethylene carbon monoxide) copolymer. A tilt crack, forming a 60° angle with the vertical boundary, extends from the left boundary to the interior of the sample. The length of the crack is 33 mm, and the end of the fracture inside the sample is at 45 mm in the vertical direction. The bottom boundary of the sample is fixed, while a small velocity of 0.01 m/s is applied at the top boundary. Figure 11d shows the fracturing simulation result at the moment of material failure. The crack, even though tilted at the initial state, horizontally propagates to the right boundary of the sample. The result is consistent with the simulation result in^22^ (Fig. 4b within^22^). Furthermore, our simulation result is in good agreement with the physics experiment results in^45^, where the real fracture propagation direction is  −28 ± 1. 5° (note that this is a value based on their experiment configuration; our simulation result is equivalently  −30° in their configuration).

In the third experiment, we simulate compression-shear fracturing with our in-house phase-field solver in a horizontally slim sample under vertical uniaxial pressures. This test example aims to mimic the experiment conducted by^46^. More specifically, we aim to mimic their sample named S-60-60 as listed in their Table 2. The sample, as shown in Fig. 11e, is 140 mm in height and 70 mm in width. The sample is discretized with *N*_horizontal_ × *N*_vertical_ = 140 × 280 regular rectangle spectral elements with a grid size of 5 × 10^−4^ m. The characteristic fracture width is *l*_0_ = 20 × 10^−4^ m. According to^46^, the upper half of the sample has a Young’s modulus *E* = 4.35 GPa, a Poisson’s ratio *ν* = 0.21, and a density *ρ* = 2, 182 kg/m^3^, while the lower half of the sample has *E* = 5.35 GPa, *ν* = 0.17, and *ρ* = 2, 355 kg/m^3^. In addition, we set *G*_*c*_ = 200 J/m^2^, mimicking common granite rock. Two tilt cracks, both with a length of 15 mm and a tilt angle of 60^°^, are at the center of the sample, with a separation of 22 mm. The boundary conditions applied at the top and bottom boundaries are pressures in the form of opposite velocity of 0.1 m/s along the vertical direction. The compression boundary conditions are expected to induce shear fracturing within the sample^46^. Figure 11f displays the simulation result generated with our phase-field solver for this sample at the moment of failure, where the two separate cracks connect with each other and extend to sample boundaries due to shearing. It is clear that our simulation result is in close correlation with the real experiment photo of rock sample failure displayed in Fig. 7j and k of^46^, especially regarding the dominant cracking path photographed in the experiment. It is important to notice that our phase-field solver does not generate the wing cracks and other secondary fractures that are observed by by^46^. However, these types of fractures are not dominant cracks in the real physics experiment. Improvement on the formulation of the history energy function to more accurately capture compression-shear strain energy concentration and evolution may improve the fidelity and accuracy of our solver in such cases.

The above three simulation results verify the efficacy and accuracy of our phase-field solver for simulating fracture nucleation and propagation from very small scale (1 mm) to large scale (100 m) under different boundary conditions (tensile and compression-shearing) and within different types of materials. This indicates that our phase-field solver provides reliable fracturing simulation results for the materials mentioned in Section 6.

### Combined finite discrete-element method

The simulation software used in this work, HOSS, includes a robust suite of benchmarks and validation studies, which we do not detail here as they are thoroughly documented elsewhere^25^. However, we do expand upon the common instances and corrective measures we account for in our analysis. These common instances are directly related to the numerical instability of the problem that is important to consider for materials undergoing fracturing, fragmentation, and contact interactions.

**Fig. 12:**
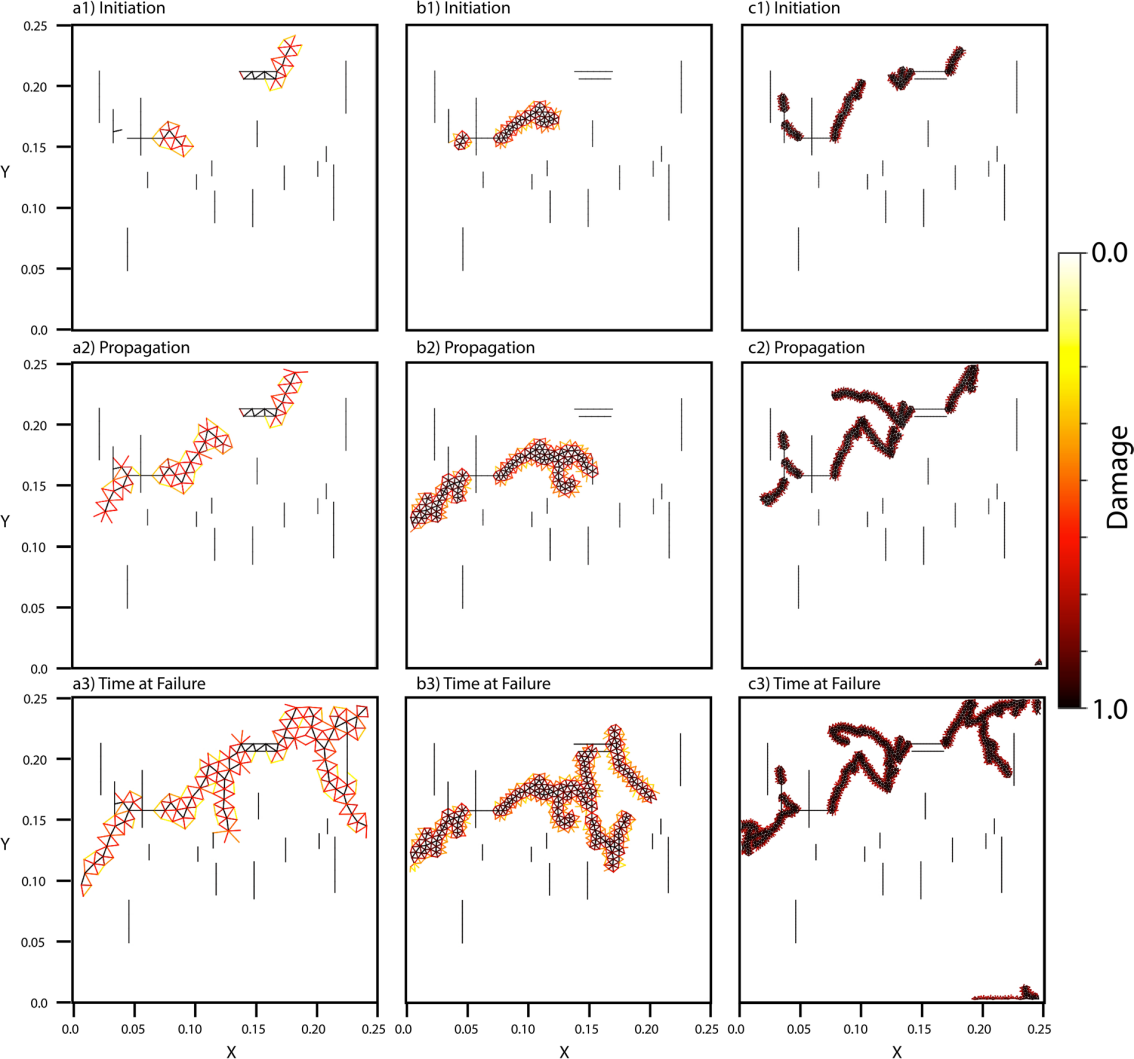
HOSS fracture example for same initial fracture configuration and material at time to failure with different mesh sizes. Each damaged cell edge is superficially extended in a Gaussian distribution about itself such that 2*σ* ≈ 1*ℓ* where *ℓ* is the characteristic element length. The distribution shows areas of most intense fracturing where *d* = 1.0 while simultaneously visualizing the mesh sizing. (**a1**–**a3**) is a mesh size  × 2 the mesh size used for our simulations. (**b1**–**b3**) is the mesh size used for our simulations ( ≈ 3 ⋅ 10^−3^ m). (**c1**–**c3**) is $\frac{1}{2}$ the mesh size used for our simulations. The bulk behavior across all domains is relatively similar, but the sensitivity on fracture propagation is dependent on the mesh once strain localization initiates. A detailed mesh-objective analysis remains beyond the scope of current dataset.

With any meshing method that incorporates an explicit time step there must be careful consideration of the critical time step based on the Courant condition. If the time step is too large numerical instability and divergence will occur. We appropriately account for this with our small time stepping 1 ⋅ 10^−8^ s. The critical time step is dependent on the mesh size with an increasing mesh allowing for a larger critical time step, but a loss of fidelity in the fracture patterns. Fracture-based finite element formulations are known to exhibit sensitivity to the underlying mesh once strain localization initiates^47,48^. For example, HOSS accurately assesses fracture coalescence by capturing distinct mixed-mode fracture processes when compared with experiment^48^, but will not match the observed fracture pattern exactly due to the mesh sensitivity. This behavior is intrinsic to damage-based representations of fracture and arising from the fracture being modeled as separation along predefined element edges, governed by cohesive laws. In practice, the bulk material response, rather than the exact fracture path, is often used to assess mesh convergence^47^. Accordingly, we include a qualitative figure demonstrating that the bulk damage evolution remains spatially consistent across different mesh sizes, indicating that the chosen discretization provides an effective convergence for the purposes of this study (Fig. 12). As the focus of the present work is on the comparative fracture behavior and material responses rather than numerical regularization, a detailed mesh-objective analysis remains beyond the scope of current dataset.

Another important consideration for FDEM problems is high-frequency oscillations. These oscillations are a direct result of small elements experiencing excessive numerical noise. During the meshing process there are sometimes combinations of fracture patterns that produce excessively small elements. These instances create numerical instability and divergence as previously explained. We apply a check of the mesh prior to our batch submissions which ignores these spurious simulations prior to running. Furthermore, there is a damping term introduced for discrete elements called Munjiza viscosity that targets numerical artifacts while leaving the real wave (energy) propagation largely unaffected^26,35^.

Due to the large number of files running in batch across multiple HPC systems there were a small handful of instances where simulations resulted in corrupted files. Since we had to post-process each HOSS simulation using the procedure detailed in Section 6, we have already assessed every simulation’s accessibility by removing those that would error when attempting to open. By post-processing the data we have ensured that there are no corrupt files or missing fractures and have also opened every file individually to assess the reliability of its contents.

## Usage Notes

The repository contains simulation output data (phase-field-based and HOSS-based simulations) organized into compressed .tar.gz archives of HDF5 (.h5) files. The data are grouped under five different material directories for the phase-field data (aluminum, PBX, shale, steel, and tungsten), and three material directories for the HOSS data (PBX, shale, and tungsten). We have provided a Python script named “extract_h5.py” in the original data repository (see https://huggingface.co/datasets/smartFRACs/material_fracturing^43^), that shows how to download and manipulate sample records from the datasets.

Listing 1 Python code to download and load the data into a dictionary and print its constituent objects. 

## Data Availability

The code and data for this project is available online^43^.
